# Duration of Spent Mushroom Substrate Return Affects Microbial Assembly and Nitrogen Metabolism to Promote Functional Stabilization in Rice–Mushroom Crop Rotation Systems

**DOI:** 10.3390/microorganisms14061251

**Published:** 2026-06-02

**Authors:** Yihong Yue, Yu Jiang, Yuchen Zhang, Tingting Xiao, Haibo Hao, Qian Wang, Zongjun Tong, Jinjing Zhang, Hui Chen

**Affiliations:** 1 National Engineering Research Center of Edible Fungi, Key Laboratory of Edible Fungi Resources and Utilization (South), Ministry of Agriculture, Institute of Edible Fungi, Shanghai Academy of Agricultural Sciences, Shanghai 201403, China; yihongyue@saas.sh.cn (Y.Y.); zyc0216@saas.sh.cn (Y.Z.); xiaotingting@saas.sh.cn (T.X.); hhb199232@163.com (H.H.); wq-15309@163.com (Q.W.); ttzjun1@163.com (Z.T.); 2 State Key Laboratory of Environmental Criteria and Risk Assessment, State Environmental Protection Key Laboratory of Simulation and Control of Groundwater Pollution, Chinese Research Academy of Environmental Sciences, Beijing 100012, China; jiangyu@craes.org.cn

**Keywords:** community assembly, nitrogen cycling, paddy soil, return duration, spent mushroom substrate (SMS)

## Abstract

Spent mushroom substrate (SMS) return is a vital strategy for agricultural waste recycling and soil fertility improvement, yet its ecological impacts of duration remain poorly understood. This study employed metagenomic sequencing to explore soil fertility, microbial dynamics, and nitrogen cycling across different SMS return durations (0, 1, and 3 years) within rice–mushroom crop rotation systems. Soil nutrients (organic matter, total nitrogen, total phosphorus) initially decreased and then increased throughout the rice growth cycle. The one-year return (y1) induced early nutrient depletion, whereas the three-year return (y3) significantly enhanced late-stage nutrient accumulation. With increasing duration, bacterial and archaeal assembly shifted from stochastic toward deterministic processes, while fungal diversity and stochasticity decreased continuously. Co-occurrence network analysis demonstrated that SMS return increased network complexity and intercommunity competition. This transition was accompanied by a functional shift in keystone taxa from those responsive to exogenous organic matter in y1 to those mediating nitrogen fixation, anammox, and sulfur metabolism in y3. Nitrogen cycling in y1 increased potential N_2_O emission risks through nirS upregulation and nosZ downregulation, whereas y3 mitigated inorganic nitrogen loss by upregulating gene abundances of ammonia assimilation, nitrification, and DNRA genes. Notably, the structure of nitrogen-cycling genes fluctuated in y1 but was resilient to y0 levels in y3. These findings demonstrated that while initial SMS return triggered ecological fluctuations and environmental risks, continuous return (y3) achieved functional stability by reshaping microbial niches. This study highlights the importance of SMS return duration in balancing soil fertility enhancement with environmental risk mitigation in sustainable paddy ecosystems.

## 1. Introduction

With the rapid advancement of circular agriculture, spent mushroom substrate (SMS) has emerged as a promising bioorganic fertilizer resource for soil amendment. As the world’s largest producer of edible fungi, China generates over 60 million tons of SMS annually, posing a critical challenge for its disposal [1]. SMS is characterized by its abundance of lignocellulose, residual fungal mycelia, amino acids, and mineral nutrients. Its loose and porous texture effectively improves soil structure and alleviates compaction [2,3]. Numerous studies have shown that direct SMS incorporation enhances soil physicochemical properties, stimulates enzymatic activities, increases organic carbon and total nitrogen storage, and improves soil fertility [4]. However, owing to its high mineral and organic contents, improper application of SMS may lead to imbalances in soil salinity and nutrients, as well as exacerbated greenhouse gas emissions [1]. Therefore, it is essential to assess the ecological responses to SMS inputs to develop effective guidelines for its application.

Soil microorganisms serve as sensitive indicators for evaluating the ecological functions of farmland. The input of exogenous organic fertilizers provides abundant carbon and nitrogen, alleviating nutrient limitations for indigenous microbes while expanding ecological niches. This, in turn, drives microbial succession and alters community diversity [5,6]. Studies have shown that microbial community responses depend largely on the type of organic fertilizer applied. Long-term livestock manure application significantly increases soil nitrogen and organic carbon, driving shifts in microbial communities from oligotrophic to copiotrophic groups capable of decomposing complex organic compounds. This promotes key taxa such as Bacillales, Gaiellales, and Pezizales, while inhibiting potential fungal pathogens [7,8]. Conversely, excessive application can reduce bacterial diversity and network stability in rice-oilseed rape rotation [9]. Crop straw, rich in labile carbon, regulates soil temperature and moisture, enhancing microbial proliferation during initial incorporation [10,11]. While specific taxa capable of degrading recalcitrant carbon sources typically dominate early on [12], the gradual depletion of easily degradable substances (such as proteins and carbohydrates) and the accumulation of resistant compounds (such as lignin and aromatic hydrocarbons) increase the difficulty of nutrient acquisition, leading to corresponding shifts in community structure [13]. Biochar, with its high porosity, large surface area, and stable aromatic carbon structure, improves soil water retention, aeration, and cation exchange capacity, thereby altering microbial habitats and substrate availability [14,15,16]. Unlike straw, which enhances the abundances of Proteobacteria and Bacteroidota, biochar often reduces the relative abundances of Ascomycota and Chytridiomycota due to its inert carbon nature and impact on enzymatic activities [17]. Compared with these conventional amendments, SMS uniquely combines lignocellulosic residues, nitrogen-containing fungal biomass, and mineral nutrients, which may lead to asynchronous carbon and nitrogen release during decomposition and generate distinct microbial ecological effects [18], resulting in significant ecological differences. However, the dynamic changes in microbial communities following SMS incorporation and their driving mechanisms remain poorly understood.

In rice–mushroom crop rotation systems, SMS return is not a discrete one-time input but a repeated process of ecological disturbance, adaptation, and stabilization. By releasing labile carbon and nitrogen substrates, SMS incorporation triggers niche fluctuations during the initial years. In our previous study, we characterized interannual shifts in microbial taxonomic diversity and soil enzymatic activities [19]. However, the mechanisms driving microbial community assembly and their functional potential remain unclear. Most research has focused on the immediate effects of a single SMS application on microbial biomass, often overlooking the interannual dynamics of microbial assembly and metabolic traits during the early stages of successive incorporation [20]. Given the characteristic high C/N ratio of SMS [21], continuous inputs are likely to alter soil nitrogen transformation pathways. We hypothesized that initial SMS return would enhance stochastic microbial assembly and increase the genomic potential for nitrogen loss, whereas continuous return would strengthen deterministic filtering and promote nitrogen-retention pathways, thereby contributing to functional stabilization. Therefore, this study aimed to investigate how different durations of SMS return regulate microbial community assembly, ecological network stability, and nitrogen cycling functions in rice–mushroom rotation systems, providing a theoretical basis for optimizing SMS management and improving the sustainability of rice agriculture.

## 2. Materials and Methods

### 2.1. Study Site, Experimental Design and Sample Collection

The field experiment was conducted from 2022–2024 at the experimental station in Xuanqiao Town, Pudong New District, Shanghai, China (31°07′ N, 121°47′ E), employing a rice–mushroom rotation system. The cultivated strain Stropharia rugosoannulata DQ-1 (CGMCC5.2211) was sown annually in November and harvested by the following April. After harvest, SMSs were incorporated into the soil through in situ rotary tillage, followed by rice cultivation (variety: Huzao 193) from May to October. Specifically, the remaining spent mushroom substrate was evenly distributed across the field plots and incorporated into the topsoil (0–20 cm) before rice transplantation. The SMS used in this experiment originated from the same cultivation system and fungal strain each year to minimize variability among treatments. Three treatments were applied in this experiment: y0, control without SMS return; y1, one-year SMS return; and y3, three-year SMS return. Treatments were laid out in a split-plot, randomized complete block design in triplicate. Each plot was 20 m in length and 15 m in width.

Soil samples were collected during the rice growth stages of tillering (A), jointing (B), flowering pollination (C) and maturing (D). Five soil cores (5 cm in diameter and 0–20 cm in depth) were collected per plot and mixed as a composite sample. Three replicate samples were collected per treatment. After sieving through 2 mm mesh, the fresh soils were divided into two portions: one was subjected to air-drying for physicochemical measurements, and the other was stored at −80 °C for subsequent DNA extraction.

### 2.2. Characterization of Soil Physicochemical Variables

Soil total nitrogen (TN) was quantified using an elemental autoanalyzer (Elemental Analyzer System Vario MACRO cube, Germany; standard deviation < 0.20% rel) [22]. Total phosphorus (TP) and total potassium (TK) were analyzed via the molybdenum blue and flame photometric methods, respectively, following digestion with H2SO4 and HClO4 [23]. Soil organic matter (SOM) was measured using the potassium dichromate oxidation method, while available nitrogen (AN) was determined by the continuous alkali-hydrolyzed reduction diffusion method. Available phosphorus (AP) was assessed via the molybdenum–antimony anti-spectrophotometric method, and available potassium (AK) was determined by flame spectrophotometry after extraction with 1 M ammonium acetate. Ammonia nitrogen (NH_4_^+^–N) and nitrate nitrogen (NO_3_^−^–N) contents were quantified via the indophenol blue colorimetry method and ultraviolet spectrophotometric detection, respectively. Soil moisture content was determined using the drying method at 105 °C for 24 h. All measurements were performed in triplicate.

### 2.3. DNA Extraction, Metagenome Sequencing and Gene Analysis

Total genomic DNA was extracted from paddy soil samples using the E.Z.N.A.^®^ Soil DNA Kit (Omega Biotek, Norcross, GA, USA) according to the manufacturer’s instructions and fragmented to approximately 400 bp using a Covaris M220 (Gene Company Limited, Shanghai, China). Agarose gel electrophoresis and Qubit 3.0 fluorometric quantification were used to evaluate the integrity and concentration of the extracted DNA. DNA library was constructed using the NEXTFLEX Rapid DNA Seq Kit (Bioo Scientific, Austin, TX, USA), and the quality and fragment size distribution of the library were assessed using an Agilent 2100 Bioanalyzer to ensure high-quality sequencing data. Sequenced was performed on an Illumina NovaSeq 6000 platform (Illumina Inc., San Diego, CA, USA) at Majorbio Bio-Pharm Technology Co., Ltd. (Shanghai, China). After quality control and adapter trimming by fastp (v0.21.0, https://github.com/OpenGene/fastp, accessed on 1 December 2024), clean reads from each sample were assembled into contigs using Megahit (v1.2.9, https://github.com/voutcn/megahit, accessed on 1 December 2024), and open reading frames (ORFs) were predicted using MetaGene (http://metagene.cb.k.u-tokyo.ac.jp/, accessed on 1 December 2024). CD-HIT (v4.8.181; http://www.bioinformatics.org/cd-hit/, accessed on 1 December 2024) was subsequently used to cluster ORFs with ≥95% identity and >90% coverage, generating a non-redundant gene catalog. The high-quality reads of each sample were mapped to the gene catalog at 95% identity using SOAPaligner (http://soap.genomics.org.cn/, accessed on 1 December 2024) to evaluate gene abundance in paddy soil samples. BLASTP (v2.2.28, http://blast.ncbi.nlm.nih.gov/Blast.cgi, accessed on 1 December 2024) was used to taxonomically annotate genes by aligning the non-redundant genes against the NCBI and NR databases (e-value < 10^−5^). Functional annotation was performed using BLASTP against the Kyoto Encyclopedia of Genes and Genomes (KEGG) database (e-value < 10^−5^). Nitrogen cycling genes and their associated taxonomic hosts were specifically extracted based on KEGG nitrogen metabolism pathways.

### 2.4. Modeling Neutral Community Assembly

To assess the relative contributions of deterministic and stochastic processes, a neutral community model (NCM) was applied to predict the relationship between species occurrence frequency and abundance in R software (version 4.12) [24]. The R2 value indicated the goodness-of-fit to the neutral model, with higher values reflecting a greater influence of stochastic processes on community assembly. The parameter N described the metacommunity size, defined as the total abundance of all species or functions within each sample. The migration rate at the community level was quantified by the parameter m, with lower values suggesting diffusion limitation within the community. Normalized stochasticity ratio (NST) analysis was employed to quantitatively evaluate ecological stochasticity based on a null model framework. The NST index was calculated through 1000 randomizations using the “NST” R package. A 50% threshold was used to differentiate between deterministic assembly (<50%) and stochastic assembly (>50%).

### 2.5. Co-Occurrence Network Construction and Keystone Identification

To explore the interactions among archaeal, bacterial and fungal communities, co-occurrence network analysis was conducted at the genus level (relative abundance > 0.1%) based on a correlation matrix in Cytoscape (version 3.8.2) [25]. Network visualization and topology analysis were carried out using Gephi (version 0.10.1). Each node represented a genus, and edges represented significant correlations between nodes (Spearman’s |ρ| > 0.6, *p* < 0.01). Node degree reflected the number of direct connections to a node, while the average degree indicated the overall correlation strength within the network. Keystone microbes within the network were identified using within-module connectivity (Zi) and among-module connectivity (Pi) analyses [26]. Nodes were sorted into four subcategories as follows: module hubs (highly connected nodes within modules; Zi > 2.5 and Pi < 0.62), connectors (nodes connecting modules; Zi < 2.5 and Pi > 0.62), network hubs (highly connected nodes within the entire network; Zi > 2.5 and Pi > 0.62), and peripherals (nodes with few outside connections; Zi < 2.5 and Pi < 0.62). Typically, the first three subcategories were defined as keystone taxa.

### 2.6. Statistical Analyses

One-way analysis of variance (ANOVA) followed by Duncan’s multiple range test was performed to determine significant differences among treatments at *p* < 0.05. Statistical analyses of archaeal, bacterial and fungal communities under different durations of SMS return were conducted using SPSS software (version 21.0). Microbial alpha-diversity indices, including Shannon, Chao1, ACE, and Simpson indices, were calculated based on the relative abundance matrix using the “vegan” package in R software (version 4.3.1). Heatmaps were generated in R software (ver. 4.0.2) with the “pheatmap” package. The Mantel test was applied to measure the effects of soil properties on microbial structures and nitrogen functional genes in R software with the “vegan” package. Euclidean distance was used to calculate the dissimilarity between environmental variables, and the Bray–Curtis distance was used to characterize microbial community composition differences. Principal coordinate analysis (PCoA) based on the Bray–Curtis distance matrix was performed to evaluate differences in microbial community composition among treatments using the “vegan” package in R software. These two matrices were then correlated to evaluate the relationship between environmental and microbial community dissimilarity.

## 3. Results

### 3.1. Dynamic Changes in Soil Physicochemical Characteristics

**Figure 1:**
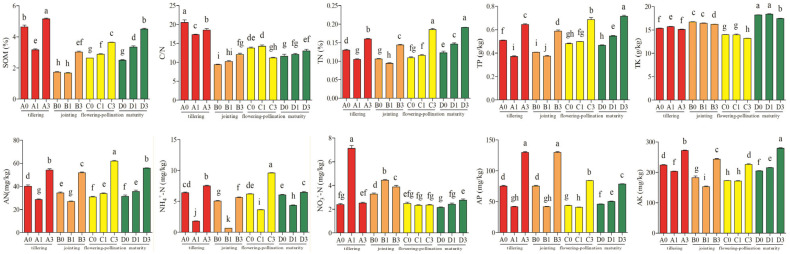
Soil physicochemical properties across four rice growth stages under different durations of spent mushroom substrate (SMS) return. A, B, C and D indicate the different rice growth stages at the tillering, jointing, flowering pollination, and maturity stages, respectively. 0, 1 and 3 indicate the different return durations with non-returning control, one-year return, and three-year return, respectively. SOM, soil organic matter; C/N, carbon–nitrogen ratio; TN, total nitrogen; TP, total phosphorus; TK, total potassium; AN, available nitrogen; NH_4_^+^–N, ammonia nitrogen; NO_3_^−^–N, nitrate nitrogen; AP, available phosphorus; and AK, available potassium. Different lowercase letters above the bars indicate significant differences among treatments (*p* < 0.05, Duncan’s multiple range test).

The duration of SMS return significantly influenced the dynamic variation in soil fertility across the four rice growth stages (Figure 1). Soil SOM, C/N, TN, TP, and NH_4_^+^–N in all treatments generally exhibited a “decline–recovery” pattern throughout the rice growth cycle, reaching the lowest levels at the jointing stage and subsequently recovering during the flowering–pollination and maturity stages. A pronounced cumulative effect of SMS return on soil fertility was observed. During the tillering and jointing stages, the three-year SMS return treatment (y3) maintained the highest SOM, TN, TP, AN, NH_4_^+^–N, AP, and AK contents, whereas the one-year return treatment (y1) showed the lowest values (*p* < 0.05). In contrast, soil NO_3_^−^–N exhibited an opposite trend (*p* < 0.05). During the flowering–pollination and maturity stages, the concentrations of SOM, TN, TP, AN, NH_4_^+^–N, AP, and AK in y3 remained significantly higher than those in y0 and y1 (*p* < 0.05). Among these variables, SOM and AN progressively increased with the duration of SMS return. The soil C/N ratio reached its maximum at the tillering stage in all treatments and remained significantly higher in y3 than in y0 during the jointing and maturity stages (*p* < 0.05). In addition, soil TK peaked at the maturity stage, whereas the y3 treatment consistently showed lower TK levels than y0 and y1 during the later growth stages (*p* < 0.05). These results indicate that y1 represents a transitional phase of nutrient fluctuation, while y3 stabilizes and enhances the nutrient pool.

### 3.2. Effects of SMS Return on Microbial Diversity and Composition

**Figure 2:**
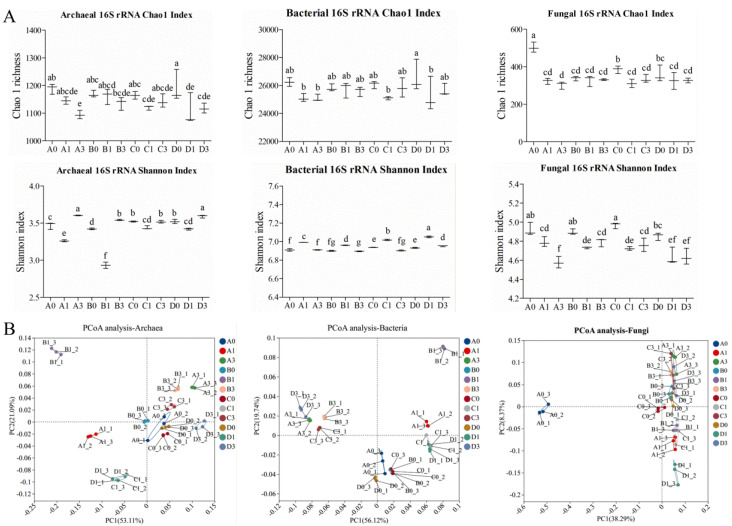
Soil microbial α diversity (**A**) and β diversity (**B**) levels across the four rice growth stages under the different durations of SMS return. For α diversity, different lowercase letters above the bars indicate significant differences among the different treatments (*p* < 0.05, Duncan’s multiple range test). For β diversity, principal coordinate analysis (PCoA) based on Bray–Curtis distances is shown.

SMS return significantly altered the soil microbial α diversity (Figure 2A). Overall, exogenous SMS input reduced microbial richness, particularly during the maturity stage. In terms of diversity, the archaeal Shannon index significantly was significantly higher in y3 compared to y0, while y1 exhibited the lowest values (*p* < 0.05). Conversely, the bacterial Shannon index peaked in y1 (*p* < 0.05), significantly surpassing both y0 and y3. In contrast, the fungal Shannon index was significantly higher in y0 compared to both y1 and y3 throughout the rice growth period (*p* < 0.05). Principal coordinate analysis (PCoA) revealed distinct shifts in the β diversity of soil microbial communities across different SMS return durations (Figure 2B). With respect to both archaea and bacteria, y1 samples clustered independently and showed significant separation from y0 and y3, indicating an unstable transitional state where the initial input of SMS disrupted the original community equilibrium. With increased duration, y3 samples exhibited tighter clustering, signifying a recovery of structural stability and a transition toward a more robust community state. In contrast, the fungal community showed minimal variation in β diversity across treatments, despite a significant decline in α diversity. These findings suggest that SMS return primarily exerts an ecological filtering effect on specific fungal taxa, rather than inducing wholesale compositional turnover, resulting in a community that is less responsive to growth-stage fluctuations.

After quality filtering, a total of 1.65 billion high-quality sequences were obtained from 36 samples, which were classified into 33 phyla and 1559 genera of archaea, 161 phyla and 4167 genera of bacteria, and 10 phyla and 960 genera of fungi. Significant differences in the relative abundances of the dominant microbial phyla (top 20) were observed across the different durations of SMS return (Figure S1). In the archaeal community, Euryarchaeota and Nitrososphaerota predominated across all the treatments, collectively accounting for over 74.38% of the total abundance (Figure S1A). During the tillering and jointing stages, Euryarchaeota abundance was significantly lower in y1 compared to both y0 and y3 (*p* < 0.05), while Nitrososphaerota showed the opposite trend. In the bacterial community, the dominant phyla were Pseudomonadota (19.64–24.57%), Chloroflexota (12.63–17.43%), and Actinomycetota (10.31–16.50%) (Figure S1B). The relative abundances of Actinomycetota, Acidobacteriota, and Bacteroidota in y3 were significantly greater than those in y0 and y1 (*p* < 0.05). Conversely, the abundances of Thermodesulfobacteriota and Candidatus Rokubacteria peaked in y1 but were at their lowest in y3. In the fungal community, the predominant phyla were Ascomycota, Mucoromycota, and Basidiomycota (Figure S1C). Notably, Basidiomycota dominated (68.37%) at the tillering stage in y0, while Ascomycota was predominant in all the other treatments, with relative abundances ranging from 41.65% to 50.74%. Except for the tillering stage, fungal composition variations across growth stages were minimal. As SMS return duration increased, the abundance of Ascomycota tended to increase. Excluding the tillering stage of y0, Mucoromycota reached its highest abundance in y1, while Basidiomycota remained significantly more abundant in y0 treatment than in the SMS-returning treatment.

### 3.3. Environmental Factors and Ecological Processes Driving Microbial Community Assembly

**Figure 3:**
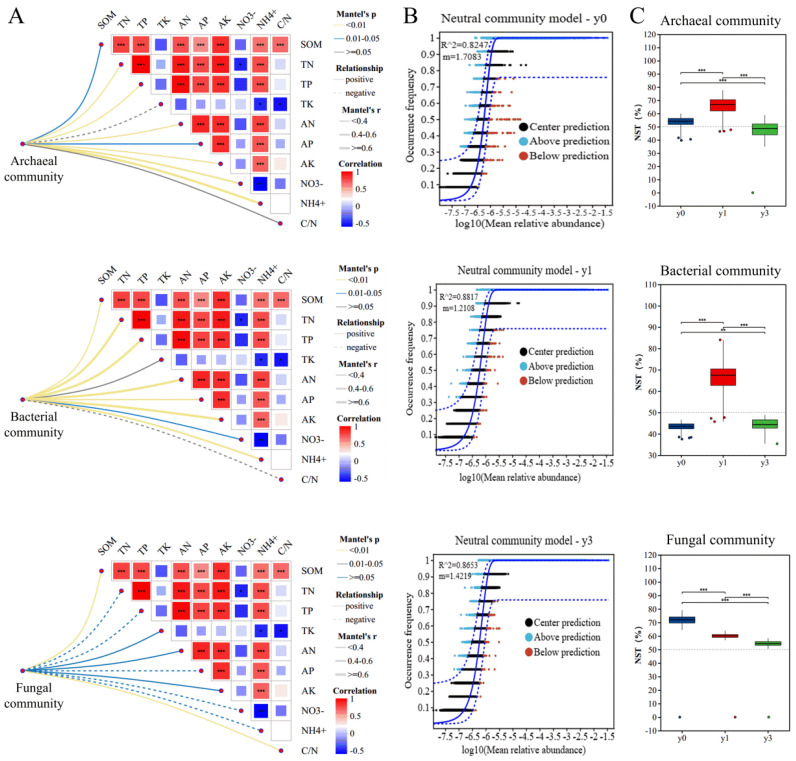
Environmental factors and ecological processes driving microbial community assembly across four rice growth stages under different durations of SMS return. (**A**), Mantel’s r values are indicated by the edge width, while the statistical significance is denoted by the edge color. Pairwise correlations of environmental variables are depicted with a color gradient reflecting Spearman’s correlation coefficient. (**B**), Fit of the neutral community model (NCM) for community assembly. The solid blue lines indicate the best fit to the NCM, and the dashed blue lines represent 95% confidence intervals around the model prediction. Species that occur more or less frequently than those predicted by the NCM are shown in different colors. m indicates the immigration rate, and R2 indicates the overall model fit. (**C**), Comparison of the normalized stochasticity ratio (NST) indices of archaeal, bacterial and fungal communities under different return durations. y0, y1 and y3 indicate the different returning durations with non-returning control, one-year returning, and three-year returning. SOM, soil organic matter; C/N, carbon–nitrogen ratio; TN, total nitrogen; TP, total phosphorus; TK, total potassium; AN, available nitrogen; NH_4_^+^–N, ammonia nitrogen; NO_3_^−^–N, nitrate nitrogen; AP, available phosphorus; and AK, available potassium. *, *p* < 0.05, **, *p* < 0.01, ***, *p* < 0.001.

The results of the Mantel test further revealed the key environmental factors driving microbial community structure (Figure 3A). Both archaeal and bacterial communities were significantly influenced by various nutrient variables, including SOM, TN, TP, AN, AP, AK, NO_3_^−^–N, and NH_4_^+^–N (*p* < 0.01 or *p* < 0.05). The structural shifts in these communities were clearly driven by the multiple nutrient inputs introduced via SMS amendment. However, the fungal community remained relatively unaffected by most available nutrients, with SOM being the primary factor influencing its composition (*p* < 0.01).

A neutral community model (NCM) was employed to assess the relationships between the occurrence frequencies of soil microbial species and their relative abundance variations (Figure 3B). The goodness of fit (R^2^) remained high across all the treatments (0.8247–0.8817), indicating that stochasticity played a predominant role in shaping community diversity. Higher R^2^ values in y1 and y3 compared to y0 suggested that the substantial nutrient input from SMS alleviated environmental selection pressure, thereby enhancing the contribution of stochastic processes to community assembly. Moreover, migration rates (m values) in y1 and y3 were significantly lower than those in y0, indicating that SMS imposed a strong dispersal limitation on microbial species, particularly in y1. The normalized stochasticity ratio (NST) was used to quantify the relative contributions of ecological processes to the assembly of archaeal, bacterial, and fungal communities (Figure 3C). In the archaeal community, stochastic processes predominated in y1, while community assembly in y3 began to shift toward deterministic selection. The bacterial community exhibited a similar dynamic, with strong stochasticity in y1 and deterministic processes predominating in both y0 and y3. In fungal communities, the contribution of stochasticity decreased progressively with increasing duration, indicating an intensifying environmental selection pressure over time.

### 3.4. Co-Occurrence Network of the Microbial Community

**Figure 4:**
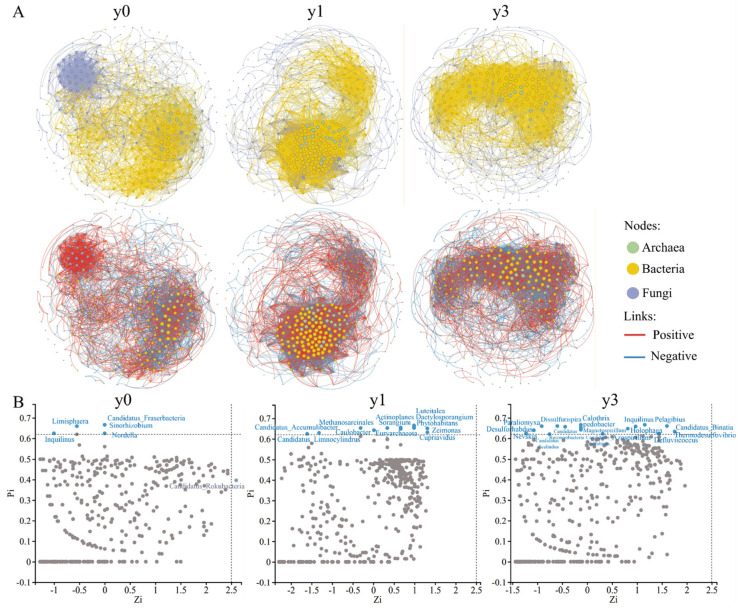
Effects of SMS return on microbial cooccurrence networks and keystone taxa identification. (**A**), Cooccurrence network of soil microbial communities at the genus level under different return durations. Each node represents a genus whose relative abundance exceeds 0.1%. Edges represent significant correlations (Spearman’s |ρ > 0.6|, FDR-corrected *p* < 0.01). The size of each node is proportional to the degree. Red and blue edges indicate positive and negative interactions, respectively. (**B**), Zi–Pi plot identifying the topological roles of microbial genera. All the genera are sorted into four subcategories as follows: peripherals (Zi < 2.5 and Pi < 0.62), connectors (Pi > 0.62), module hubs (Zi > 2.5), and network hubs (Zi > 2.5 and Pi > 0.62). The gray nodes indicate insignificant genera of peripherals, the blue nodes indicate key genera of connectors, and the purple nodes indicate key genera of module hubs.

To explore the potential interactions among soil microorganisms under SMS return conditions, three co-occurrence networks encompassing archaeal, bacterial, and fungal communities were constructed at the genus level (relative abundance > 0.1%) (Figure 4A). In y0, fungal nodes accounted for a high amount (43.36%) and exhibited distinct modularity, with fungal genera forming tightly clustered, relatively independent modules. Following SMS return, the boundaries between different kingdoms became blurred, suggesting that SMS input significantly stimulated synergistic cross-taxon interactions. Topological analysis (Table 1) revealed that the networks comprised 354–369 nodes, with bacterial and archaeal proportions increasing slightly over time, while fungal proportions decreased from 43.36% to 39.83%. Compared to y0, the number of edges in y1 increased substantially from 3310 to 8679, with the average degree rising from 17.94 to 47.426 and graph density increasing from 0.049 to 0.13, indicating that SMS return during the first year significantly intensified ecological connectivity. Although these parameters decreased in y3, they remained higher than in y0. Notably, the average path length was shortest in y3 (3.192), suggesting that three years of continuous return enhanced the community’s rapid response capacity to SMS input. Furthermore, modularity reached its minimum in y1 and maximum in y0, indicating that SMS return weakened the initially highly segmented modular structure and enhanced community integration. In terms of interspecies correlations, y1 and y3 exhibited greater proportions of negative links compared to y0, suggesting that SMS input intensified niche competition or antagonistic interactions.

The topological role of nodes was determined by calculating within-module connectivity (Zi) and among-module connectivity (Pi) values to identify potential keystone genera (Figure 4B). Most nodes (94.92–98.37%) were classified as peripherals. In y0, one module hub and five connectors were identified, predominantly belonging to the bacterial phyla Pseudomonadota (Sinorhizobium, Nordella, and Inquilinus) and Verrucomicrobiota (Limisphaera) (Table S1). In y1, 12 keystone nodes connecting the connectors were identified, including archaeal genera from Euryarchaeota (g__unclassified_p__Euryarchaeota and g__unclassified_o__Methanosarcinales) alongside bacterial genera from Pseudomonadota (Zeimonas, Cupriavidus, and Caulobacter), Myxococcota (Sorangium), Actinomycetota (Phytohabitans, Dactylosporangium, and Actinoplanes), and Actinobacteriota (Luteitalea). In y3, 18 genera that commonly existed in connectors were considered potential keystones, which included seven bacterial phyla, namely, Pseudomonadota (Pelagibius, Nevskia, Magnetospirillum, Inquilinus, Defluviicoccus, and Azospirillum), Nitrospirota (Thermodesulfovibrio, Dissulfurispira, and Candidatus_Sulfobium), Bacteroidota (Pedobacter), Myxococcota (Paraliomyxa), Acidobacteriota (Holophaga), Thermodesulfobacteriota (Desulforhabdus), Planctomycetota (Candidatus_Scalindua) and Cyanobacteriot (Calothrix). These results demonstrate that SMS return enhances the number of key nodes that connect connectors, drastically altering keystone genera in the network, with the abundances of potential keystones rising continuously over time.

### 3.5. Response of Nitrogen Metabolism Pathways and Functional Genes to SMS Return

**Figure 5:**
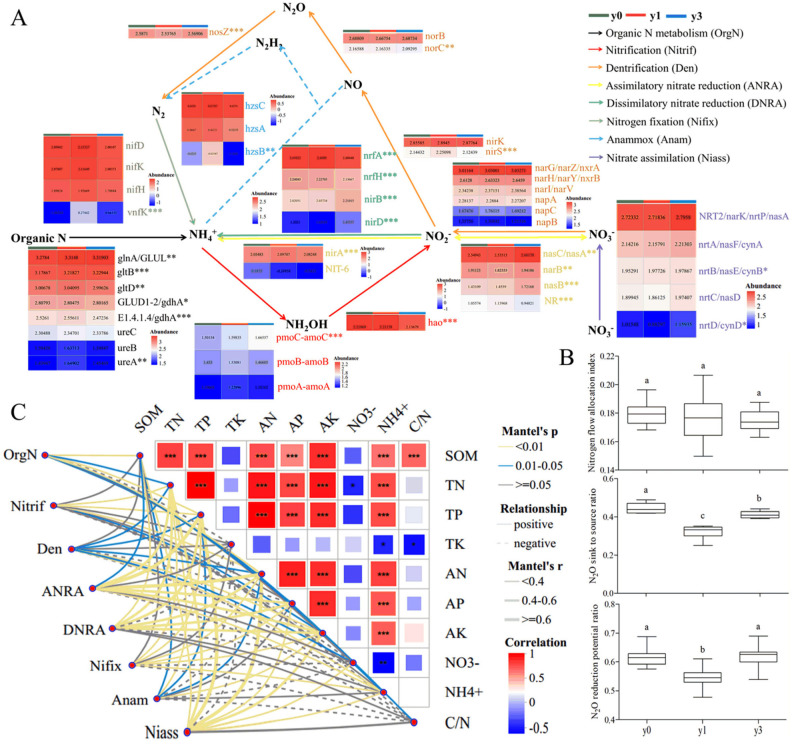
Responses of soil nitrogen cycling functional genes and their driving factors under different durations of SMS return. (**A**), Heatmap illustrating the abundance of functional genes involved in nitrogen cycling. The mean values and standard errors are presented (*n* = 12). Genes with significant changes in abundance are marked with asterisks (*, *p* < 0.05; **, *p* < 0.01; ***, *p* < 0.001) on the basis of log10-transformed abundance data. (**B**), Box plots of the impact of SMS return on soil nitrogen turnover pathways. Different lowercase letters indicate significant differences among treatments (*p* < 0.05). (**C**), Mantel tests correlating eight nitrogen cycling processes (determined by the Bray–Curtis distance) with environmental variables (determined by the Euclidean distance). Mantel’s r values are indicated by the edge width, while the statistical significance is denoted by the edge color. Pairwise correlations of environmental variables are depicted with a color gradient reflecting Spearman’s correlation coefficient. OrgN, organic nitrogen metabolism; Nitrif, nitrification; Den, denitrification; ANRA, assimilatory nitrate reduction; DNRA, dissimilatory nitrate reduction; Nifix, nitrogen fixation; Anam, annamox; and Niass, nitrate assimilation.

To comprehensively understand nitrogen metabolism under different SMS return durations, functional genes associated with eight major nitrogen cycling pathways were analyzed (Figure 5A). Regarding organic nitrogen metabolism (OrgN), the abundances of gltB (*p* < 0.001), glnA (*p* < 0.01) and gltD (*p* < 0.01) exhibited a clear upward trend with increasing SMS return duration, suggesting an increase in microbial ammonia assimilation potential over time. This enhanced microbial capacity facilitates the conversion of inorganic nitrogen into biomass. In nitrification (Nitrif), the key ammonia oxidation gene amoC was significantly upregulated as the duration of return increased (*p* < 0.001), reflecting a promotion of ammonia oxidation efficiency in y3. In denitrification (Den), nirS and nosZ were highly significantly different across treatments (*p* < 0.001). Specifically, nirS was most abundant in y1, while nosZ abundance decreased, suggesting a potentially increased risk of N_2_O emission during the first year of SMS. However, the abundances of both genes stabilized by y3. Concurrently, the gene abundances of the nirB and nirD genes, which were involved in dissimilatory nitrate reduction to ammonium (DNRA), significantly increased in y3. Since DNRA reduced nitrate to ammonium rather than gaseous nitrogen, y3 was more conducive to soil nitrogen conservation. In DNRA and nitrate assimilation (Niass), genes such as nrtB/nasE/cynB, nasB, and nirA were significantly upregulated following SMS return, demonstrating intensified microbial assimilation and utilization of nitrate, which helped reduce the risk of inorganic nitrogen loss. Regarding nitrogen fixation (Nifix), vnfK abundance significantly increased in y1, indicating that initial return activated the vanadium-dependent nitrogen fixation pathway as a sensitive response to environmental shifts. The impacts of SMS return on nitrogen cycling and environmental risks were further evaluated (Figure 5B). The N_2_O sink-to-source ratio and N_2_O reduction potential were significantly lower in y1 than those in y0 and y3 (*p* < 0.05), confirming an increased risk of N_2_O emission during the initial year of SMS return. Nevertheless, no significant differences in the nitrogen flow allocation index were observed across treatments, suggesting that SMS return had not yet disrupted the overall balance between nitrogen retention and loss.

The results of the Mantel test (Figure 5C) revealed that TN, TP, AN, and AK were the most critical regulatory factors and were significantly correlated with functional genes across all eight nitrogen cycling processes. In contrast, the C/N ratio was primarily correlated only with OrgN. Specifically, OrgN was significantly positively correlated with almost all the environmental factors (Mantel r < 0.4; *p* < 0.05), except for NO_3_^−^–N. Nitrif was predominantly positively correlated with TN, TP, AN, and AK (Mantel r < 0.4; *p* < 0.05), while Den was significantly positively correlated with NH_4_^+^–N, AK, NO_3_^−^–N, TP, AN, and TN (Mantel r < 0.4; *p* < 0.05). ANRA and Niass were most strongly driven by AN and TP (Mantel r > 0.6, *p* < 0.01), followed by TN and AK (Mantel r = 0.4–0.6, *p* < 0.01). Similarly, DNRA was the most closely associated with AN (Mantel r = 0.568; *p* = 0.001), followed by TP, AK, AP, and TN (Mantel r = 0.4–0.6; *p* < 0.01). Nifix was positively correlated with AP, AN, TP, NH4+, AK, and TN (Mantel r < 0.4, *p* < 0.01), whereas Anam was driven mainly by AK, TN, TP, SOM, and AN (Mantel r < 0.4, *p* < 0.05).

To further elucidate the relationships between functional genes and nitrogen-cycling microorganisms, the taxonomic hosts of key genes were analyzed (Figure S2). In OrgN, unclassified d_p_Candidatus-Rokubacteria and Nitrospira were identified as shared hosts. Specifically, for urea, the contributions of Nitrospira and unclassified_f__Nitrososphaeraceae were significantly greater in y1 than in y0 and y3, consistent with the peak abundance of ureA observed in y1. In Nitrif, Nitrospira was the dominant host of pmoC–amoC in both y0 (26.77%) and y3 (29.99%), while its contribution markedly decreased to 8.9% in y1, where unclassified d_f_Nitrososphaeraceae (35.89%) and Candidatus–Nitrosocosmos (27.88%) predominated, indicating that AOA played a key role in ammonia oxidation during y1. Conversely, y3 exhibited a synergistic increase between AOA and AOB. In Den, both nosZ and norC shared similar core hosts, such as Anaeromyxobacter, indicating the presence of high-efficiency denitrifying microbes. For nirS, Anaerolina was the dominant host in y0 and y3, while Thiobacillus emerged as the primary contributor in y1, which was accompanied by a notable decrease in the contributions of Anaerolinea and Bradyrhizobium. For ANRA and DNRA, the contributions of Nocardioids to genes such as nasC/nasA, nirB, and nirD increased progressively with increasing SMS return duration, with Nocardioides dominating the core host role, particularly for nasB. Similarly, for Nifix and Anam, genes such as vnfK and hzsB showed significant taxonomic contributions from DPANN_group_archaeon and Candidatus_Brocadia, respectively. Across all nitrogen cycling pathways, the composition of dominant host genera remained highly consistent among treatments, with functional shifts being primarily driven by variations in the relative contribution of shared hosts.

## 4. Discussion

### 4.1. Differential Response Patterns of Microbial Taxa Under SMS Return

Soil nutrients serve as fundamental indicators of soil quality. In this study, continuous SMS return (y3) significantly enhanced the SOM, TN, TP, and available nutrient contents, demonstrating the potential of SMS to reconstruct the soil organic matter pool and increase soil fertility, consistent with previous reports [27,28]. In contrast, during the tillering and jointing stages of the first year (y1), most nutrients, except for NO_3_^−^–N, declined significantly, suggesting that the substantial influx of exogenous carbon initially triggered a strong positive priming effect, thereby accelerating the turnover of native soil nutrients. As the primary drivers of soil ecosystems, microorganisms respond to nutrient inputs in ways that directly regulate ecological functions. SMS incorporation led to an overall reduction in microbial richness (Figure 2), likely due to the selective pressures exerted by the complex lignocellulosic components and secondary metabolites released during SMS decomposition, which promoted distinct phylogenetic selection [29,30]. Additionally, the large input of readily available carbon and nitrogen sources likely stimulated the rapid proliferation of copiotrophic microorganisms, thereby intensifying competitive exclusion and reducing niche heterogeneity within the microbial community. Long-term nutrient enrichment may further simplify community composition by allowing dominant functional groups to occupy major ecological niches. Meanwhile, changes in soil physicochemical properties, including nutrient availability, oxygen microenvironments, and potentially pH conditions, could also contribute to the restructuring of microbial diversity. Furthermore, SMS decomposition releases a wide range of organic compounds and fungal-derived metabolites, which may selectively stimulate specific microbial guilds involved in lignocellulose degradation, nitrification, DNRA, and denitrification pathways. These biochemical processes collectively influence nutrient turnover, oxygen microenvironments, and soil acidity, thereby reshaping microbial community structure and ecological networks. The potential role of pH-mediated environmental filtering may further contribute to shifts in microbial diversity and community assembly under continuous SMS return. In y1, bacteria dominated the decomposition niches of labile carbon, leveraging their rapid growth and high nutrient affinity [31,32]. Soil with high microbial diversity is often associated with accelerated nutrient turnover rates [33], which strongly supports the coupling observed between increased bacterial diversity and nutrient depletion in y1. Moreover, the initial SMS input significantly increased the abundance of Nitrososphaerota (Figure S1), which suppressed other archaeal taxa through competitive exclusion, thereby reducing archaeal diversity. This heightened nitrification activity is further corroborated by the depleted NH_4_^+^–N and increased NO_3_^−^–N in the rice tillering and jointing stages of y1.

As the duration of return increased (y3), the soil ecosystem gradually stabilized. Accumulation of lignocellulose degradation products and the formation of stable microaerophilic environments facilitated the optimization of specialized archaeal niches, significantly enhancing archaeal diversity [34]. Among bacteria, the relative abundance of Actinomycetota increased significantly (Figure S1). This phenomenon could be attributed to the continuous accumulation of cellulose and lignin from prolonged SMS incorporation, which activated the ability of Actinomycetota to secrete β-glucosidase and xylanase to degrade these complex carbon sources [35]. Furthermore, the enrichment of Acidobacteria and Bacteroidota in y3 suggested that continuous return ensured a stable nutrient supply. The decline in y3 bacterial diversity to the y0 level reflected the re-equilibration of community structure as resources reached a steady state. Notably, fungal diversity continued to decline with increasing duration of return. As primary drivers of the soil carbon cycle, fungi dominate the degradation of complex lignocellulosic structures [31,36], a role confirmed by the Mantel test results showing that the fungal community was significantly correlated only with SOM (Figure 3A). However, in habitats inundated with abundant labile organic substrates, the rapid proliferation of specific fungal taxa established niche dominance, subsequently simplifying community structure and reducing diversity [37]. Moreover, in nutrient-enriched environments, fungi, which often favor oligotrophic growth, are at a disadvantage in terms of competition for space and resource protection against copiotrophic bacteria [38]. These characteristics could explain the observed decreases in the proportions of fungal nodes within the co-occurrence networks as the duration of SMS return increased (Table 1).

### 4.2. Dynamic Shifts in Community Assembly and Network Stability

SMS incorporation significantly increased the stochastic processes in the soil microbiome compared with y0 (Figure 3B). Exogenous organic matter likely alleviated environmental selection pressure and expanded ecological niches by increasing spatial heterogeneity [39]. Such stochastic-driven high phylogenetic diversity is generally considered to increase community adaptability during periods of environmental fluctuation [40]. Furthermore, the relatively low migration rates (m values) in y1 and y3 revealed a pronounced dispersal limitation induced by SMS return, potentially due to altered soil pore structure or microhabitat connectivity, resulting in localized physical isolation and restricted random dispersal. Notably, the assembly mechanisms exhibited distinct taxon-specific responses to the duration of SMS return. In y1, both archaeal and bacterial communities were dominated by stochastic processes, reflecting that intense disturbance and heavy nutrient loading during the initial stage of return temporarily masked the intensity of environmental filtering, prompting the communities to maintain stability through enhanced stochastic succession [41,42]. In y3, as the habitat gradually stabilized, the continuous accumulation of specific organic components induced niche-based environmental filtering, shifting the assembly mechanism toward determinism. This transition could be closely linked to fluctuations in community diversity. Xun et al. [43] reported that environmental selection increased in low-diversity bacterial communities, which contributed to the dominance of deterministic processes. This theory aligned with our observation that bacterial diversity decreased significantly in y3, which coincided with the transition toward deterministic assembly. In contrast, the fungal community exhibited a steady decline in stochasticity with increasing duration of return. This phenomenon could be attributed to the strong directional selection of substrates for fungi as lignocellulose decomposers, which aligned with our finding that the fungal community was significantly driven by SOM. Importantly, while NST analysis indicated a deterministic shift in specific taxa in y3, the NCM maintained a consistently high goodness of fit (R2 > 0.8) at the whole-community level. These results collectively demonstrated that stochastic processes remained the core mechanism shaping the soil microbial community in this paddy field, giving the soil ecosystem a robust buffering capacity and stability.

Co-occurrence network analysis revealed that the network in y1 exhibited significantly greater complexity, with increased total edges, average degree, graph density, and average clustering coefficient relative to y0. This indicated that the abundant nutrient supply during the initial stage stimulated intensive microbial interactions. Simultaneously, the input of SMSs increased the proportion of negative correlations, suggesting an intensification of niche competition or antagonistic interactions among soil microorganisms. In nutrient-rich habitats, niche overlap intensifies, and competition often replaces cooperation as the dominant ecological force [44]. The increased pressure of species competition can effectively suppress the cascading diffusion of internal perturbations within the network, maintaining the robustness of the ecosystem during periods of drastic fluctuation [45]. In contrast, the reduction in the average path length and the recovery of the proportion of positive correlations in y3 suggested that the community had transitioned to a highly adaptive steady state. Furthermore, SMS return significantly altered the identity of keystone genera within the network, with the number of potential keystones increasing continuously with the duration of return. In the present study, 12 rare species were identified as keystones in y1. Members of the order Methanosarcinales play key roles in methane production in paddy fields and are involved in multiple metabolic pathways, including acetoclastic, hydrogenotrophic, and methylotrophic methanogenesis [46]. Taxa belonging to Myxococcota (Sorangium) facilitate the decomposition of complex polysaccharides by secreting various extracellular enzymes [47], whereas microbes of the genus Actinobacteriota (Actinoplanes) play critical roles in degrading highly lignified tissues in SMSs and have been classified as probiotic entities for plants [48]. In y3, Azospirillum has a high nitrogen fixation capacity and can convert atmospheric N2 into bioavailable ammonium, alleviating the nitrogen limitation associated with SMS decomposition [49]. Candidatus_Scalindua is a genus for anaerobic ammonium oxidation (Anammox) that contributes to nitrogen removal in paddy ecosystems [50], whereas Magnetospirillum participates in denitrification and utilizes nitrate as an electron acceptor under anaerobic conditions [51]. Members of Desulfobacterota (Desulforhabdus) engage in dissimilatory sulfate reduction within anaerobic niches to obtain energy, and their competition with methanogens for electron donors potentially suppresses methane emissions [52]. Both Thermodesulfovibrio and Dissulfurispira belong to the Nitrospirota phylum, with the former being a globally widespread genus primarily associated with anaerobic niches in thermal habitats, while the latter can fix CO_2_ via the Wood–Ljungdahl pathway and perform sulfur disproportionation [53]. These results collectively demonstrate that SMS incorporation fosters a complex and robust ecological network by promoting stochastic community assembly and strengthening interactions among keystone taxa. This successional transition, shifting from taxa capable of rapidly responding to exogenous organic matter in y1 to functional groups mediating nitrogen fixation, Anammox, and sulfur metabolism regulation in y3, not only maintains the structural stability of the paddy ecosystem but also significantly enhances its ability to synergistically regulate multielement cycling.

### 4.3. Functional Responses of Nitrogen Cycling to SMS Return

Nitrogen is the primary limiting nutrient in agroecosystems, with its speciation and bioavailability largely regulated by microbial activity [54]. In y1, the abundance of the denitrification gene nirS peaked, whereas nosZ significantly decreased, leading to a reduced N_2_O sink-to-source ratio. Notably, while metagenomic analysis quantified the genetic potential for N_2_O emissions rather than in situ flux, this genomic pattern indicated an elevated risk of nitrogen loss during the initial phase. This fluctuation stemmed from transient shifts in host structure, notably the transition of the primary nirS contributor from Anaerolinea to Thiobacillus. Given the sulfur-rich nature of SMS, the dominance of Thiobacillus suggested a sulfur-driven denitrification pathway that likely led to the heightened N_2_O emissions in the early stage. With respect to nitrification, AOA (Nitrososphaeraceae) dominated in y1, likely resulting from localized oxygen depletion induced by heterotrophic respiration following the substantial input of organic carbon, allowing AOA—which are more tolerant of low-oxygen conditions and possess a higher ammonia affinity—to outcompete AOB [55,56]. Furthermore, while several key genes for nitrogen fixation (nifD/K/H) and anaerobic ammonium oxidation (Anammox, hzsA/C) remained relatively stable, the contributions of the specific responsive genes vnfK and hzsB were restricted to single taxa (DPANN_group_archaeon and Candidatus Brocadia, respectively). Such low functional redundancy implied that the specific supplementary mechanisms of these two pathways for coping with initial habitat fluctuations were highly vulnerable [57]. In y3, a significant shift in the ammonia assimilation pathway from gdhA to gltB/glnA indicated that continuous SMS return prompted the microbiome to adopt a high-affinity nitrogen capture strategy, maximizing the conversion of inorganic nitrogen into microbial biomass in a habitat where resource turnover had stabilized, thereby effectively reducing the risk of nitrogen leaching [58]. Concurrently, the alternative response of nirB/D over nrfA/H within the DNRA pathway was closely associated with the increased contribution of the core host Nocardioides, further enhancing nitrogen retention capacity [59]. Crucially, the dynamics of Nitrospira—which was depleted in the turbulent y1 phase but successfully re-established as a core host in y3—perfectly exemplified the mechanism of functional stabilization. This re-emergence signified synergistic increases in AOA and AOB, which improved the robustness of nitrification and provided a stable nitrate substrate for the DNRA pathway. Consequently, while the initial stage of SMS return maintained functionality through the recruitment of specific stress-responsive taxa, continuous return resulted in the establishment of an efficient nitrogen-retention system, achieving synergistic optimization of nutrient supply and environmental benefits in paddy fields.

## 5. Conclusions

This study demonstrates that the duration of SMS return significantly affects the soil microenvironment and nitrogen cycling pathways in paddy fields. Although the initial incorporation (y1) provides abundant substrates, it triggers stochastic community assembly and increases the risk of N_2_O emissions. Conversely, continuous return for three years (y3) induces a shift in bacterial and archaeal assembly from stochasticity toward deterministic selection, increasing the complexity of molecular ecological networks through increased node connectivity and intercommunity competition. Furthermore, the functional roles of keystone taxa evolve from responding to exogenous organic matter in the early stage to mediating nitrogen fixation, anammox, and sulfur metabolism in the later stage, establishing a robust ecosystem with nutrient conservation. Consequently, specific mitigation strategies (e.g., nitrification inhibitors or water management) may be needed during the initial year (y1) to bridge the transition to the stable y3 stage. Overall, compared with the ecological fluctuations of the initial stage, continuous return effectively optimizes microbial functional niches, leveraging the sustained benefits of SMS to increase soil fertility and functional stabilization.

## Figures and Tables

**Table 1:** Topological properties of the microbial co-occurrence networks under the different durations of SMS return.

	y0	y1	y3
Total nodes	369	366	354
Proportion of archaea, bacteria and fungi nodes	12.74%:43.90%:43.36%	12.84%:45.90%:41.26%	14.12%:46.05%:39.83%
Total edges	3310	8679	4699
Average degree	17.94	47.426	26.548
Network Diameter	11	17	11
Graph density	0.049	0.13	0.075
Modularity	0.549	0.207	0.384
Average clustering coefficient	0.479	0.575	0.488
Average path length	3.455	3.564	3.192
Positive links	62.42%	52.91%	53.65%

## Data Availability

The metagenomic sequence data generated in this study were deposited in the NCBI Sequence Read Archive (SRA) database under the BioProject number PRJNA1413893.

## Supplementary Materials

The following supporting information can be downloaded at: https://www.mdpi.com/article/10.3390/microorganisms14061251/s1, Figure S1: Relative abundance (%) of archaeal (A), bacterial (B) and fungal (C) phyla across the four rice growth stages under different durations of SMS return. The other category represents phyla with a relative abundance of <1%; Figure S2: Contribution of microbial hosts to significantly different functional genes (*p* < 0.05) related to eight nitrogen pathways at the genus level (the top 30 genera) under different durations of SMS return. The specific taxonomic affiliations of the host genera are color coded as indicated in the legend; Table S1: Composition of keystone taxa in the co-occurrence network.
