# Supplementary material for: Duration of Spent Mushroom Substrate Return Affects Microbial Assembly and Nitrogen Metabolism to Promote Functional Stabilization in Rice–Mushroom Crop Rotation Systems
Source: Microorganisms. 2026 Jun 2;14(6):1251. doi: 10.3390/microorganisms14061251 (PMC13302999; doi:10.3390/microorganisms14061251)
Supplement: Supplementary file 1 [file microorganisms-14-01251-s001.zip › microorganisms-4346296-supplementary.pdf]

Supporting Information

Table S1 Composition of keystone taxa in the co-occurrence network.

Treatments	Hub nodes	Relative abundance (%)	Zi	Pi	Node_type	Taxonomy
y0	g__unclassified_p__Candidatus_Fraserbacteria	0.029718	0	0.66667	Connectors	Bacteria;p__Candidatus_Fraserbacteria
	g__Sinorhizobium	0.029279	0	0.66667		Bacteria;p__Pseudomonadota;c__Alphaproteobacteria;o__Hyphomicrobiales;f__Rhizobiaceae
	g__Nordella	0.02907	0	0.625		Bacteria;p__Pseudomonadota;c__Alphaproteobacteria;o__Hyphomicrobiales
	g__Limisphaera	0.076662	-0.54911	0.66		Bacteria;p__Verrucomicrobiota;c__Verrucomicrobiae;o__Limisphaerales;f__Limisphaeraceae
	g__Inquilinus	0.030631	-1.00273	0.625		Bacteria;p__Pseudomonadota;c__Alphaproteobacteria;o__Rhodospirillales;f__Rhodospirillaceae
y1	g__unclassified_p__Candidatus_Rokubacteria	3.890064	2.59567	0.39669	Module hubs	Bacteria;p__Candidatus_Rokubacteria
	g__Zeimonas	0.03198	1.31201	0.65123	Connectors	Bacteria;p__Pseudomonadota;c__Betaproteobacteria;o__Burkholderiales;f__Burkholderiaceae
	g__unclassified_p__Euryarchaeota	4.684999	0.33858	0.65306		Archaea;p__Euryarchaeota
	g__unclassified_o__Methanosarcinales	1.483873	-0.31037	0.65306		Archaea;p__Euryarchaeota;c__Methanomicrobia;o__Methanosarcinales
	g__Sorangium	0.121324	0.66306	0.64876		Bacteria;p__Myxococcota;o__Polyangiales;f__Polyangiaceae
	g__Phytohabitans	0.05283	0.98754	0.65349		Bacteria;p__Actinomycetota;c__Actinomycetes;o__Micromonosporales;f__Micromonosporaceae
	g__Luteitalea	3.051591	0.98754	0.65586		Bacteria;p__Acidobacteriota;c__Vicinamibacteria;o__Vicinamibacterales;f__Vicinamibacteraceae
	g__Dactylosporangium	0.06366	0.98754	0.66587		Bacteria;p__Actinomycetota;c__Actinomycetes;o__Micromonosporales;f__Micromonosporaceae
	g__Cupriavidus	0.103142	1.31201	0.63199		Bacteria;p__Pseudomonadota;c__Betaproteobacteria;o__Burkholderiales;f__Burkholderiaceae
	g__Caulobacter	0.035738	0.01411	0.64266		Bacteria;p__Pseudomonadota;c__Alphaproteobacteria;o__Caulobacterales;f__Caulobacteraceae
	g__Candidatus_Limnocylindrus	0.025661	-1.60828	0.625		Bacteria;p__Chloroflexota;c__Candidatus_Limnocylindria;o__Candidatus_Limnocylindrales;f__Candidatus_Limnocylindraceae
	g__Candidatus_Accumulibacter	0.1556	-1.32102	0.62942		Bacteria;p__Pseudomonadota;c__Betaproteobacteria
	g__Actinoplanes	0.129044	0.66306	0.65569		Bacteria;p__Actinomycetota;c__Actinomycetes;o__Micromonosporales;f__Micromonosporaceae
y3	g__unclassified_p__Candidatus_Roizmanbacteria	0.02695	-0.44296	0.65702	Connectors	Bacteria;p__Candidatus_Roizmanbacteria
	g__unclassified_c__Candidatus_Binatia	0.538238	1.75753	0.63327		Bacteria;p__Candidatus_Binatota;c__Candidatus_Binatia
	g__Thermodesulfovibrio	0.330232	1.75753	0.63342		Bacteria;p__Nitrospirota;c__Thermodesulfovibrionia;o__Thermodesulfovibrionales;f__Thermodesulfovibrionaceae

g__Pelagibius	0.02726	1.60036	0.66062	Bacteria;p__Pseudomonadota;c__Alphaproteobacteria;o__Hyphomicrobiales;f__Rhodovibrionaceae
g__Pedobacter	0.04888	-0.1286	0.65278	Bacteria;p__Bacteroidota;c__Sphingobacteriia;o__Sphingobacteriales;f__Sphingobacteriaceae
g__Paraliomyxa	0.201644	-0.91449	0.66	Bacteria;p__Myxococcota;c__Myxococcia;o__Myxococcales
g__Nevskia	0.070544	-1.22885	0.625	Bacteria;p__Pseudomonadota;c__Gammaproteobacteria;o__Nevskiales;f__Nevskiaceae
g__Magnetospirillum	0.045564	-0.1286	0.64266	Bacteria;p__Pseudomonadota;c__Alphaproteobacteria;o__Rhodospirillales;f__Rhodospirillaceae
g__Inquilinus	0.027456	0.97165	0.65866	Bacteria;p__Pseudomonadota;c__Alphaproteobacteria;o__Rhodospirillales;f__Rhodospirillaceae
g__Holophaga	0.068435	1.1547	0.66667	Bacteria;p__Acidobacteriota;c__Holophagae;o__Holophagales;f__Holophagaceae
g__Dissulfurispira	0.076596	-0.60013	0.66049	Bacteria;p__Nitrospirota;c__Thermodesulfovibrionia;o__Thermodesulfovibrionales;f__Dissulfurispiraceae
g__Desulforhabdus	0.059792	-1.07167	0.64	Bacteria;p__Thermodesulfobacteriota;c__Syntrophobacteria;o__Syntrophobacterales;f__Syntrophobacteraceae
g__Defluviicoccus	0.086362	1.44318	0.62268	Bacteria;p__Pseudomonadota;c__Alphaproteobacteria;o__Rhodospirillales;f__Rhodospirillaceae
g__Candidatus_Sulfobium	0.235111	0.30985	0.62281	Bacteria;p__Nitrospirota;c__Nitrospiria;o__Nitrospirales;f__Nitrospiraceae
g__Candidatus_Scalindua	0.046943	-0.75731	0.6208	Bacteria;p__Planctomycetota;c__Candidatus_Brocadiia;o__Candidatus_Brocadiales;f__Candidatus_Scalinduaceae
g__Calothrix	0.033365	-0.1286	0.66667	Bacteria;p__Cyanobacteriota;c__Cyanophyceae;o__Nostocales;f__Calotrichaceae
g__Azospirillum	0.100828	0.81447	0.64746	Bacteria;p__Pseudomonadota;c__Alphaproteobacteria;o__Rhodospirillales;f__Azospirillaceae

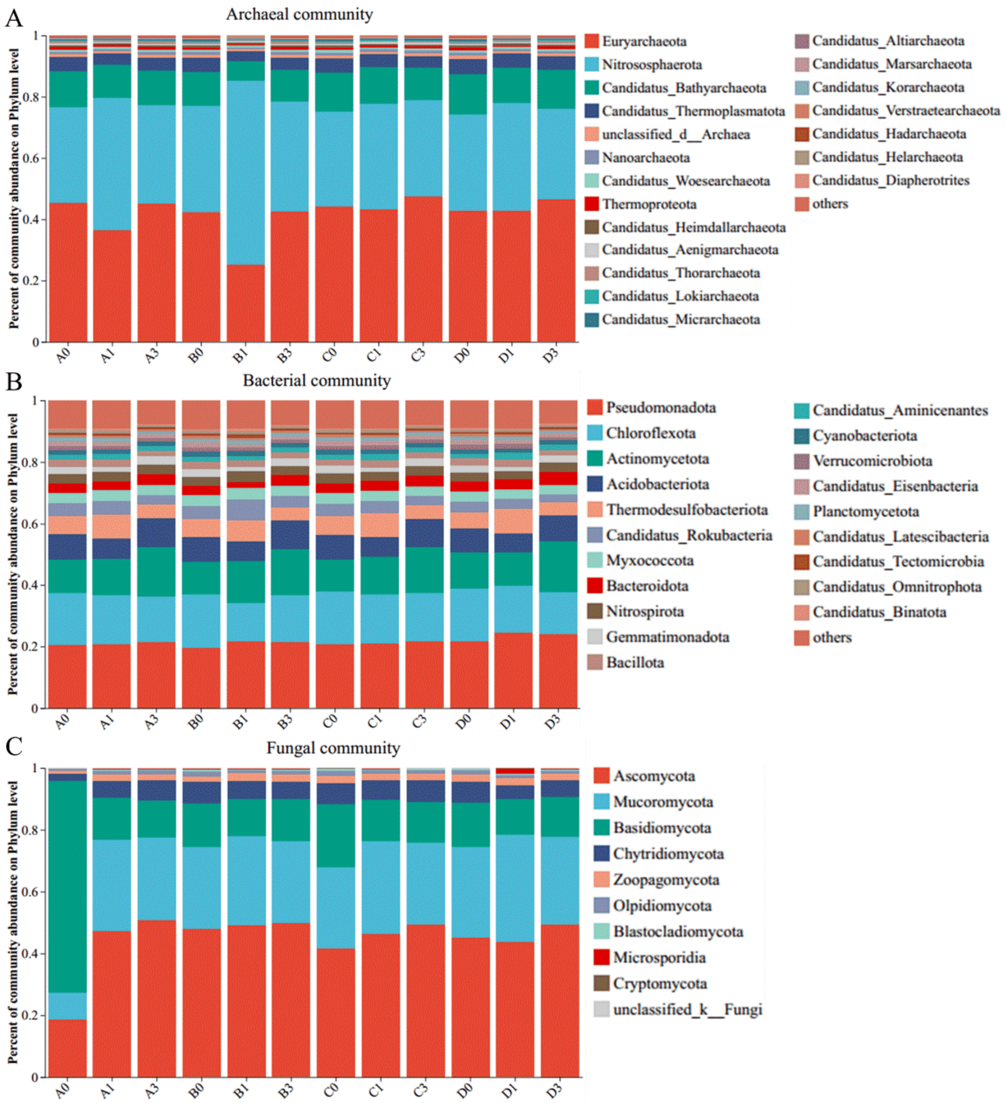

Fig. S1 Relative abundance (%) of archaeal (A), bacterial (B) and fungal (C) phyla across the four rice growth stages under different durations of SMS return. The other category represents phyla with a relative abundance of < 1%.

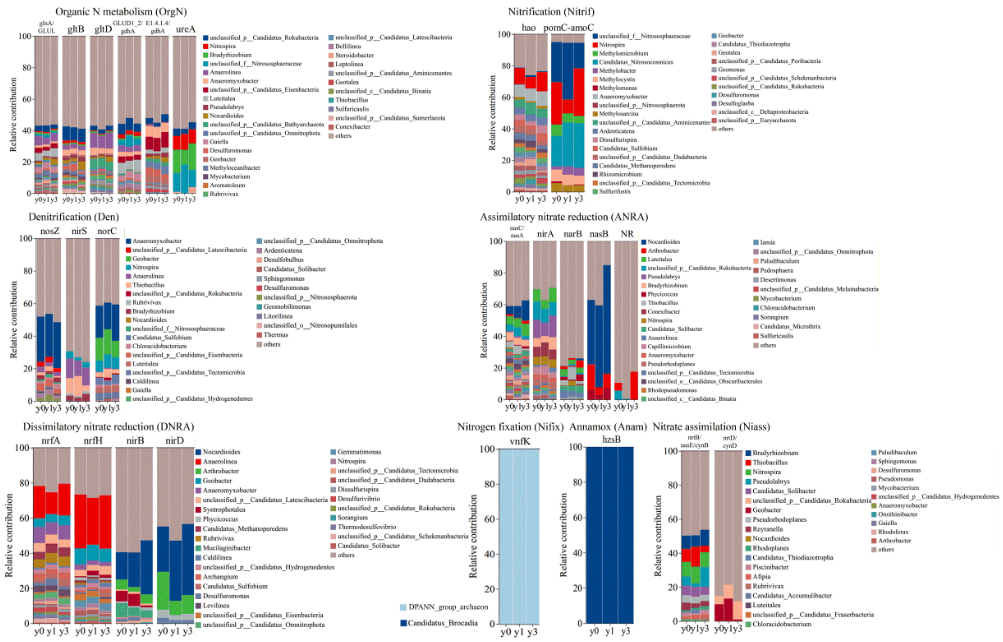

Fig. S2 Contribution of microbial hosts to significantly different functional genes ($p < 0.05$) related to eight nitrogen pathways at the genus level (the top 30 genera) under different durations of SMS return. The specific taxonomic affiliations of the host genera are color coded as indicated in the legend.
